# Viscoelastic Properties and Thermal Stability of Nanohydroxyapatite Reinforced Poly-Lactic Acid for Load Bearing Applications

**DOI:** 10.3390/molecules26195852

**Published:** 2021-09-27

**Authors:** Feven Mattews Michael, Mohammad Khalid, Gunasunderi Raju, Chantara Thevy Ratnam, Rashmi Walvekar, Nabisab Mujawar Mubarak

**Affiliations:** 1 Department of Chemistry, Alfaisal University, P.O. Box 50927, Riyadh 11533, Saudi Arabia; fevenmattews@gmail.com; 2 Department of Chemical Engineering, Faculty of Science and Engineering, University of Nottingham Malaysia, Semenyih 43500, Malaysia; 3 Graphene & Advanced 2D Materials Research Group (GAMRG), School of Engineering and Technology, Sunway University, No. 5, Jalan Universiti, Bandar Sunway, Petaling Jaya 47500, Malaysia; 4 School of Distance Education, Universiti Sains Malaysia, Gelugor 11800, Malaysia; 5 Radiation Processing Technology Division, Malaysian Nuclear Agency, Kajang 43000, Malaysia; chantaraa1@gmail.com; 6 Department of Chemical Engineering, School of Energy and Chemical Engineering, Xiamen University Malaysia, Jalan Sunsuria, Bandar Sunsuria, Sepang 43900, Malaysia; rashmi.walvekar@gmail.com; 7 Department of Chemical and Energy Engineering, Faculty of Engineering and Science, Curtin University, Miri 98009, Malaysia; mubarak.mujawar@curtin.edu.my

**Keywords:** poly-lactic acid, nanohydroxyapatite, viscoelastic properties, thermal stability, effectiveness, nanocomposites

## Abstract

We studied the reinforcing effects of treated and untreated nanohydroxyapatite (NHA) on poly-lactic acid (PLA). The NHA surface was treated with three different types of chemicals; 3-aminopropyl triethoxysilane (APTES), sodium *n*-dodecyl sulfate (SDS) and polyethylenimine (PEI). The nanocomposite samples were prepared using melt mixing techniques by blending 5 wt% untreated NHA and 5 wt% surface-treated NHA (mNHA). Based on the FESEM images, the interfacial adhesion between the mNHA filler and PLA matrix was improved upon surface treatment in the order of mNHA (APTES) > mNHA (SDS) > mNHA (PEI). As a result, the PLA-5wt%mNHA (APTES) nanocomposite showed increased viscoelastic properties such as storage modulus, damping parameter, and creep permanent deformation compared to pure PLA. Similarly, PLA-5wt%mNHA (APTES) thermal properties improved, attaining higher Tc and Tm than pure PLA, reflecting the enhanced nucleating effect of the mNHA (APTES) filler.

## 1. Introduction

The reinforcement of biodegradable polymers such as polylactic acid (PLA) with ceramics such as nanohydroxyapatite (NHA) has been the focus of many researchers, especially with respect to tissue engineering applications [1,2,3]. According to previous studies, metal-based materials have several drawbacks, including viscoelastic behaviour, weak adhesion, creep, stress shielding, and biocompatibility [4]. In addition, these metal alloys do not prompt the regeneration of the damaged bone tissue and require surface treatment in order to promote new tissue proliferation on these materials to provide total implantation [5]. Therefore, researchers are more interested in viscoelastic behaviour, which is one of the most complex issues in polymeric materials. For improving the design and fabrication of the polymer scaffoldings, exploring the viscoelastic property in detail is essential. The viscoelastic behaviour of the polymers is depicted when a polymer undergoes deformation and exhibits both viscous and elastic behaviours [6].

Aside from viscoelastic behaviour, the thermal properties of the nanocomposite, such as melting, crystallization, and glass transition temperatures, can aid in understanding the polymer’s composition. The essence and strength of interfacial bonding between the polymer and the filler contribute to most composites’ thermal stability [7]. The presence of active hydroxyl groups and the enhanced surface would improve hydrogen bonding and, therefore, thermal stability of the composite is efficiently distributed over several bonds [8].

There are a few reviews of the literature on several viscoelastic properties of PLA reinforced NHA. However, a lack of comprehensive assessment of the viscoelastic properties of all PLA reinforced fillers still remains. Therefore, this study will focus on the viscoelastic properties of PLA reinforced with NHA before and after surface treatment. The NHA was synthesized via the precipitation method, and the surface was later modified using 3-aminopropyl triethoxysilane (APTES), sodium *n*-dodecyl sulfate (SDS), and polyethylenimine (PEI). The effectiveness of NHA on the morphology, storage modulus, thermal stability, and creep behaviour of nanocomposite before and after surface modification is thoroughly investigated.

## 2. Materials

NatureWorks LLC (Boulder, CO, USA), kindly supplied the PLA (IngeoTM biopolymer 3052D). The chemicals used to produce NHA, such as di-ammonium hydrogen phosphate, calcium nitrate tetrahydrate (C.P.), ammonium solution (30%), and absolute alcohol 99.7% (denatured), were procured from LGC Scientific, Malaysia. To modify the synthesized NHA, 3-aminopropyl triethoxysilane (APTES), 99%, and polyethylenimine (PEI) were purchased from Sigma-Aldrich, Malaysia. Meanwhile, Alfa Aesar supplied the sodium *n*-dodecyl sulphate (SDS) 99% (dry wt.) and water <1.5%. All chemicals were used as purchased without any further purification.

### 2.1. Preparation of Nanocomposites

Initially, we used our optimized sono-synthesis approach to prepare NHA. Later, the synthesized NHA was surface modified using 5 wt% APTES, SDS, and PEI to produce mNHA [1]. The PLA matrix was then reinforced with the produced nanofillers (NHA and mNHA) by melt mixing for 10 min at 170 °C with a rotor speed of 100 rpm using a Haake Rheomix Polydrive R600/610 (Waltham, MA, USA). PLA was charged into the mixing chamber and melted for 2 min at 170 °C. After that, the nanofillers were added to the molten PLA and mixed for an additional 8 min. The nanofiller loading was kept constant at 5 wt% for both NHA and mNHA, and Table 1 summarizes the composition of the prepared nanocomposites. Finally, by using a tabletop RAY-RAN injection moulding machine (Warwickshire, UK) with 170 °C barrel and 90 °C mould temperatures, the nanocomposites (PLA, PLA-NHA and PLA-mNHA) were moulded into experimental specimens.

### 2.2. Dynamic Mechanical Analysis (DMA)

A dynamic mechanical analyzer (DMA, TA instrument TA01 DMA 2980, New Castle, DE, USA) was used to study the viscoelastic properties of the nanocomposites by using a dual cantilever mode. The sample size was cut at 25 × 6 × 3 mm for the studies. The storage modulus and peak of damping factor at the glass transition temperature (T_g_) of the nanocomposites were recorded between 30 and 150 °C, with a heating rate of 5 °C/min, using a frequency of 1 Hz.

The creep properties of the nanocomposites (25 × 6 × 3 mm) were determined by dual cantilever mode using DMA. The creep recovery cycles were conducted at an isothermal temperature of 70 °C (T_g_ of pure PLA). An isothermal temperature of 10 °C above and below Tg was used to evaluate the effect of temperature on creep characteristics. For 20 min at each temperature, 10% of the average tensile strength of pure PLA was applied, followed by another 20 min of recovery. A graph of static strain versus time was plotted for all nanocomposites in order to study their creep properties.

### 2.3. Thermal Stability

A Mettler Toledo TGA/DSC 1 analyzer (Columbus, OH, USA) fitted with the STAR^e^ System was used to calculate thermal weight loss, crystallization, and melting temperatures, as well as their respective heat flux with respect to the nanocomposites. The tests employed about 10 mg of the sample and were carried out in a nitrogen environment (flow rate = 30 mL/min) with temperatures ranging from 30 to 500 °C and a heating rate of 10 °C/min. T_5%_, T_10%_, T_50%_, and T_max_ were used to determine the temperature at which the nanocomposite lost 5%, 10%, 50%, and ~100% of its weight, respectively. In addition, the crystallization temperature (T_c_) and melting temperature (T_m_) are obtained from the DSC curve’s peak, and the corresponding heat flux at T_c_ and T_m_ are HF_c_ and HF_m_, respectively.

### 2.4. Field Emission Scanning Electron Microscope (FESEM)

The field emission scanning electron microscope, FESEM (FEI Quanta 400, Hillsboro, OR, USA), was used to examine the nanocomposites’ microstructure.

## 3. Results and Discussion

### 3.1. Mixing Torque

**Figure 1:**
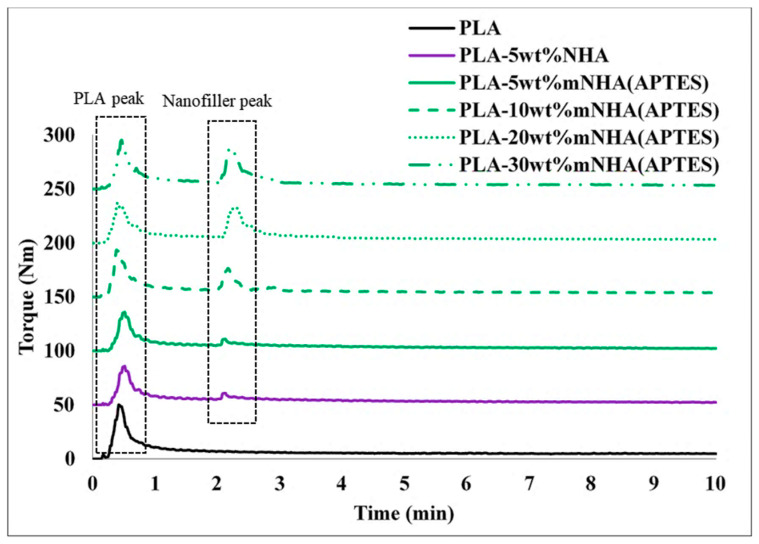
Mixing torque graph for PLA, PLA-5wt%NHA, and PLA-mNHA (APTES) nanocomposites.

Figure 1 shows the mixing torque-time curve of PLA, PLA-NHA, and PLA-mNHA (APTES) nanocomposites. As PLA was first charged into the mixing chamber, the first peak was observed at 30 s. The peak gradually dropped at about 1 min and stabilized at around 2 min. The drop in torque indicates that the PLA started to melt, while the torque stabilization signifies that the PLA has completely melted and homogenized. After the PLA mixing torque had stabilized, the nanofillers were introduced in the blend, causing the mixing torque to rise again (second peak). The increase in the second mixing torque was, however, influenced by the nanofillers’ loading. For instance, when the mNHA (APTES) loading increased beyond 10 wt%, the mixing torque increased tremendously compared to 5 wt% loading. This is due to the increased viscosity upon higher loading of mNHA (APTES). After a few seconds, the mixing torque of the nanocomposites decreased due to PLA and mNHA (APTES) melting, indicating complete mixing and homogeneity of the nanocomposite [9].

### 3.2. Surface Morphology

**Figure 2:**
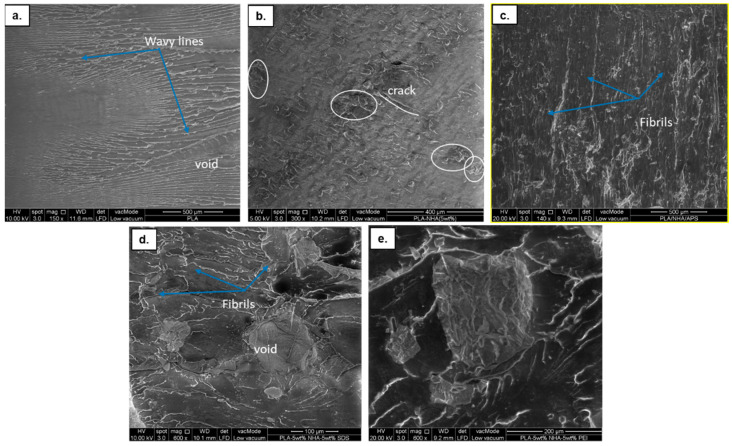
Microstructural images of (**a**) PLA; (**b**) PLA-5wt%NHA; (**c**) PLA-5wt%mNHA (APTES); (**d**) PLA-5wt%mNHA (SDS); and (**e**) PLA-5wt%mNHA (PEI).

Figure 2 shows the microstructural images of the nanocomposites in contrast to pure PLA. It is observed that the pure PLA in Figure 2a displays relatively smooth fractures, which is a typical brittle fracture behaviour. Upon the addition of 5 wt% NHA, the surface of the PLA-NHA nanocomposites showed significantly different surfaces, where the majority of fracture plane was observed with agglomerates, as pointed out with circles in Figure 2b. Furthermore, the presence of a crack path indicates that the interfacial adhesion between the PLA matrix and the NHA was poor. Figure 2c illustrates improved dispersion and interfacial adhesion between the mNHA (APTES) and the PLA matrix, with a lack of agglomeration and voids due to surface treatment. In addition, the presence of fibril suggests that the PLA-5wt%mNHA (APTES) became rigid than compared to the NHA-reinforced PLA nanocomposite. In addition, the surface modification of NHA with SDS and PEI also increased interfacial adhesion [1], which is evidenced by the lack of significant voids, as observed in Figure 2d,e, respectively. Despite this, both nanocomposites displayed the presence of agglomerates, as indicated by the circles, which could be responsible for the decrease in void fraction [10]. However, the size of the agglomerated mNHA (PEI) particles in the PLA matrix was more significant compared to mNHA (SDS). This could be due to NHA particles binding through the polyethylenimine group present in the PEI.

### 3.3. Storage Modulus

**Figure 3:**
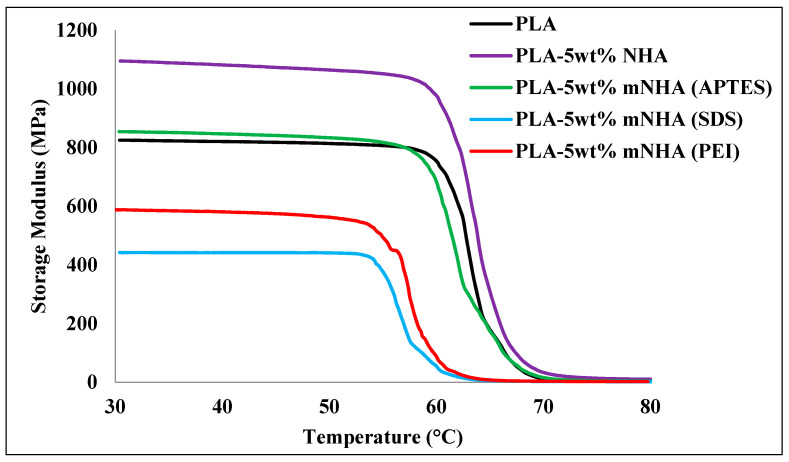
Storage modulus of the PLA, PLA-5wt%NHA, and PLA-5wt%mNHA treated with APTES, SDS, and PEI.

The storage moduli of the surface-treated nanocomposites (PLA-5wt%mNHA), untreated nanocomposite (PLA-5wt%NHA), and pure PLA are depicted in Figure 3, and Table 2 summarizes the corresponding data. For all the samples, a decrease in the storage modulus with increasing temperature was observed. This is because PLA chain mobility increases as the temperature rises, lowering the stiffness of the nanocomposites [11]. Thus, the glassy region (40 °C) occurs at the highest storage modulus, while a rapid decrease is noticed around the transition region (above 60 °C). This can also be observed from the calculated storage modulus retention percentage shown in Table 2. For instance, as the temperature increased in the transition region from 60 °C to 70 °C, the pure PLA sample recorded a significant drop of 89.7% in storage modulus, similar to the other nanocomposites.

Pure PLA has a lower storage modulus than NHA nanocomposites at all temperatures, indicating that adding nanofiller increases the stiffness of the PLA matrix by limiting PLA chain mobility. The PLA-5wt%NHA nanocomposite recorded an optimum storage modulus at the glassy and transition regions. The storage modulus of PLA-5wt%NHA nanocomposite was 28.2% higher than the storage modulus of pure PLA. However, surface treatments of NHA affect the storage modulus of nanocomposites, as evidenced by a 28% reduction in PLA-5wt% mNHA (APTES) nanocomposite compared to PLA-5wt%NHA.

On the contrary, SDS and PEI treated PLA-5wt%mNHA show a drastic decrease of 59.27% and 45.60% in storage moduli compared to PLA-5wt%NHA and pure PLA decreased by 46.16% and 28.08%, respectively. Additionally, the storage moduli of SDS and PEI reduced by 47.94% and 30.45%, respectively, compared to the PLA-5wt%mNHA (APTES) nanocomposite.

This is attributed to the improved interfacial adhesion between the mNHA (APTES) and the PLA matrix, increasing the PLA chain mobility and reducing the storage modulus. However, the PLA-5wt%mNHA (APTES) storage modulus was higher than pure PLA because the presence of the mNHA (APTES) nanofiller within the PLA matrix has restricted PLA chain mobility to a certain extent [12].

The effectiveness of the nanofillers (NHA and mNHA) on the storage modulus of the nanocomposite can be expressed by a coefficient C calculated by using Equation (1), where $E_{G}^{\prime }$ and $E_{R}^{\prime }$ denote storage moduli at the glassy (T = 40 °C) and rubbery (T = 80 °C) states, respectively. As the effectiveness of the nanofiller increases, the C value becomes lower, indicating significant reinforcement ability of the nanofiller on the polymer matrix. The calculated C values for all the nanocomposites are listed in Table 2. It can be observed that the PLA-5wt%NHA nanocomposite attained the lowest C value, which contradicts the assumptions made before about NHA not being suitable for reinforcing the PLA matrix due to poor interfacial adhesion.

(1) $$C=\frac{{\left(E_{G}^{\prime }/E_{R}^{\prime }\right)}_{\mathrm{nanocomposite}}}{{\left(E_{G}^{\prime }/E_{R}^{\prime }\right)}_{\mathrm{neat}\;\mathrm{PLA}}}$$

### 3.4. Damping Parameter

**Figure 4:**
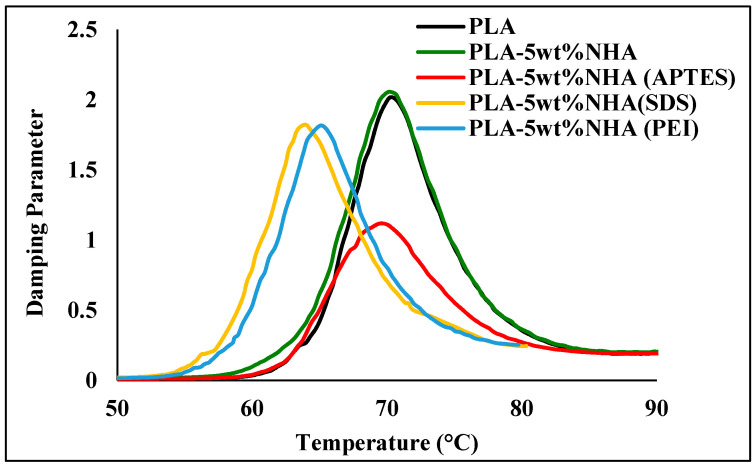
Damping parameter of the PLA, PLA-5wt%NHA, and PLA-5wt%mNHA treated with APTES, SDS, and PEI.

Figure 4 and data presented in Table 2 provide insight into the damping properties and T_g_ of the PLA-5wt%mNHA nanocomposites compared to PLA-5wt%NHA and pure PLA. In comparison to pure PLA, all of the nanocomposites had lower damping parameter peak heights. The damping properties of polymeric materials are always related to the internal resistance caused by molecular chain motions. Therefore, the lower damping properties can be attributed to the improvements in the interfacial adhesion between the nanofiller and the polymer matrix [13]. In contrast to PLA-5wt%NHA nanocomposites, the damping parameter peak height of the PLA-5wt% mNHA (APTES, SDS, and PEI) nanocomposites decreased significantly after surface treatment. This can be attributed to mNHA and PLA having better interfacial adhesion than compared to NHA and PLA. As a result, compared to PLA-5wt% NHA, the damping parameter peak height of PLA-5wt%mNHA (APTES) observed the lowest damping parameter value, decreasing by 45%. On the other hand, T_g_ of the PLA-5wt%NHA nanocomposite did not improve significantly compared to pure PLA. Although the T_g_ of PLA-5wt%mNHA nanocomposites treated with APTES showed a negligible decrease of 0.9%, PLA-5wt%mNHA nanocomposites treated with SDS and PEI showed a significant decrease of 9.3% and 7.3%, respectively, than compared to PLA-5wt% NHA nanocomposites, suggesting poor interfacial adhesion between PLA matrix and mNHA modified with SDS and PEI. This corroborates with the SEM analysis of the fractured nanocomposites samples.

### 3.5. Creep and Recovery

**Figure 5:**
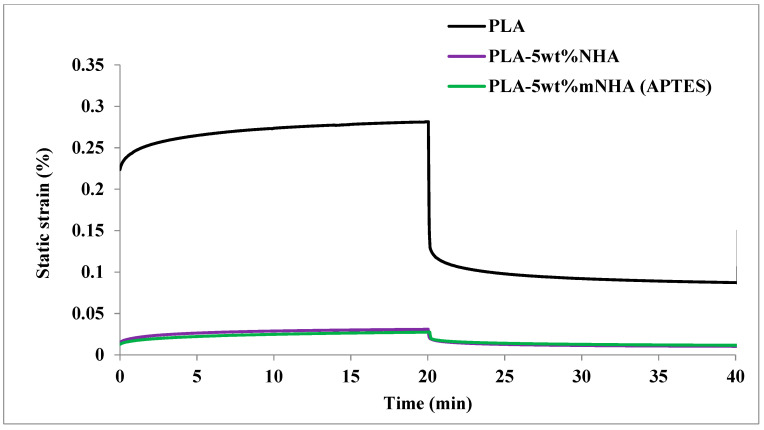
Creep and recovery curve of the PLA, nanocomposites before and after surface modification.

**Figure 6:**
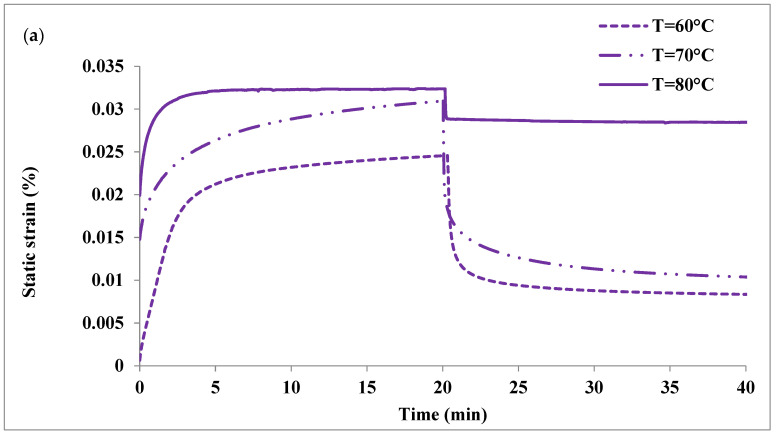

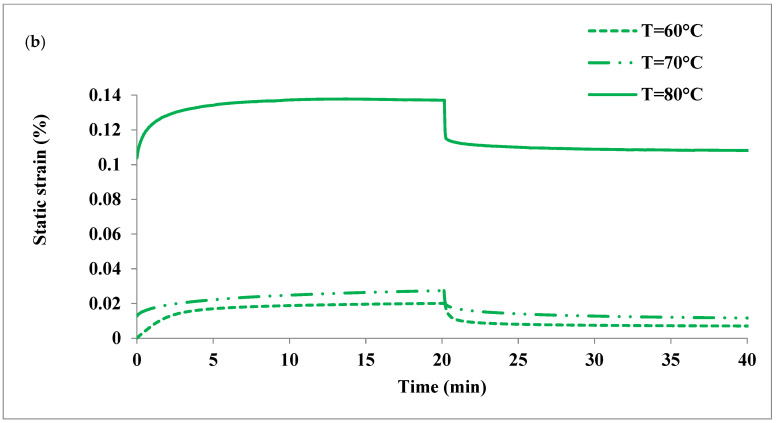
Effect of temperature on the creep and recovery curve of (**a**) PLA-5wt%NHA and (**b**) PLA-5wt%mNHA (APTES).

**Figure 7:**
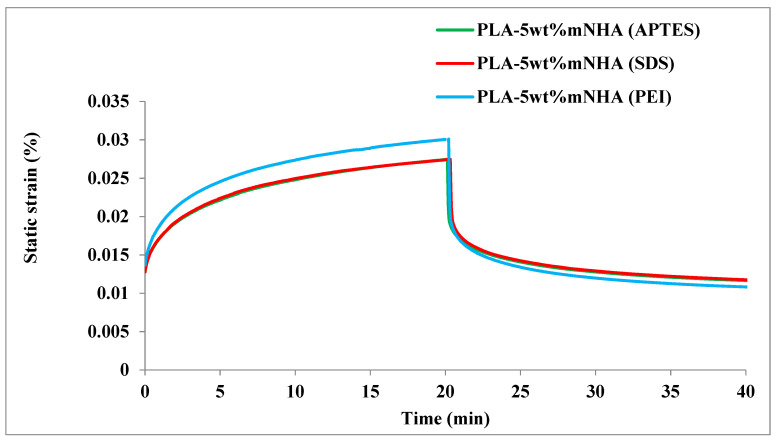
Creep and recovery curve of APTES, SDS, and PEI surface-treated PLA-5wt%mNHA nanocomposites.

In comparison to pure PLA, the creep and recovery curves of the nanocomposites are presented in Figure 5, Figure 6 and Figure 7. For this study, the pure PLA and nanocomposites were subjected to a constant static load of 0.5 N (10% of PLA’s tensile strength) for 20 min. The effect of temperature on the creep properties of PLA-5wt%NHA and PLA-5wt%mNHA (APTES) was studied. In addition, the impact of mNHA loading on creep properties was also investigated. Table 3 summarises the creep, recovery, and residual strains of nanocomposites and pure PLA. Figure 5 shows that nanocomposites displays creep behaviour in the same manner as pure PLA does. However, due to the inclusion of nanofillers, the creep and recovery strains of the nanocomposites were lower than those of the pure PLA. For instance, upon adding 5wt%NHA, the creep and recovery strains at T_g_ (70 °C) recorded a reduction of 89.1% and 89.45%, respectively, compared to pure PLA, indicating improved elasticity. In comparison to pure PLA, the PLA-5wt%NHA nanocomposite experienced considerable reduction in the residual strain of 88.1%, implying that no irreversible deformation occurred.

Meanwhile, the creep and residual strains of the PLA-5wt% mNHA (APTES) nanocomposites recorded no significant changes (less than ~1%) compared to PLA-5wt%NHA. The recovery Strain, on the other hand, experienced a 23.4% decrease; hence, based on the creep and recovery strains obtained, the optimum improved creep properties were achieved for the PLA-5wt%mNHA (APTES) nanocomposite.

The effect of surface treatment on creep properties at different temperatures is illustrated in Figure 6a,b. As expected, creep strain increased as a result of changing the temperature from 60 °C to 80 °C, the creep strain increased by 31.7% (PLA-5wt% NHA) and 537.7% (PLA-5wt%mNHA (APTES)), at t = 20 min, which is not favourable. In addition, a large permanent deformation (residual strain) was observed at T = 80 °C even though most of the strain was recovered. This demonstrates that creep properties are susceptible to temperature changes.

Figure 7 depicts a comparison of the creep properties of APTES, SDS, and PEI treated PLA-5wt%mNHA nanocomposites. Compared to PLA-5wt%mNHA (APTES), the creep properties of the SDS treated nanocomposite did not show any significant changes. Meanwhile, PEI treated PLA-5wt%mNHA nanocomposite attained higher creep strain (9.9%) and recovery strain (22.9%) and lower residual strain (7.7%) than APTES treated PLA-5wt%mNHA. The increased recoverable strain of PLA-5wt%mNHA (PEI) could be a result of the poor interfacial adhesion between the mNHA (PEI) and PLA matrices.

### 3.6. Thermal Stability

#### 3.6.1. Thermogravimetric Analysis

**Figure 8:**
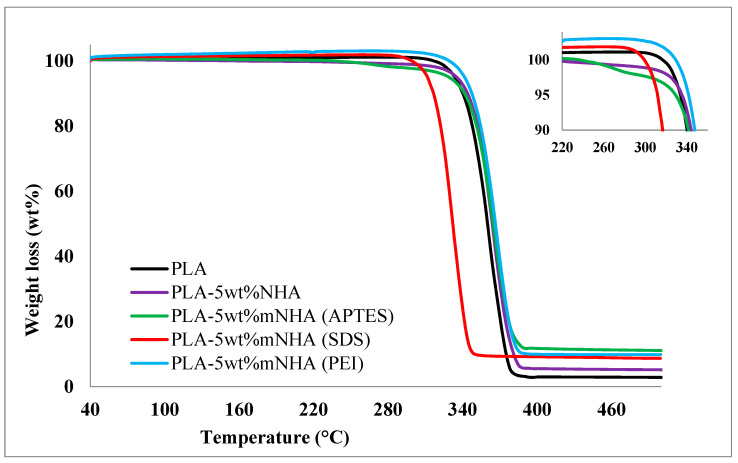
TGA thermogram for PLA, PLA-5wt%NHA, and PLA-5wt%mNHA treated with APTES, SDS, and PEI.

Figure 8 depicts the thermal stability of surface-treated (PLA-mNHA) and untreated (PLA-NHA) nanocomposites compared to pure PLA, and Table 4 summarizes the results. The TGA thermogram clearly shows that all nanocomposites display single-stage weight loss between 320 and 385 °C. The complete degradation of the pure PLA at a high temperature causes extreme weight loss. The thermal stability of the APTES and PEI-treated nanocomposites at 5wt%mNHA loading was found to improve. In contrast, the SDS-treated nanocomposites showed lower thermal stability than compared to pure PLA and PLA-5wt%NHA. Similarly, the residual weight of the nanocomposites increased with surface treatment in the order of PLA-5wt%mNHA (APTES) > PLA-5wt%mNHA (PEI) > PLA-5wt%mNHA (SDS) > PLA-5wt%NHA > PLA.

#### 3.6.2. Differential Scanning Calorimetry

**Figure 9:**
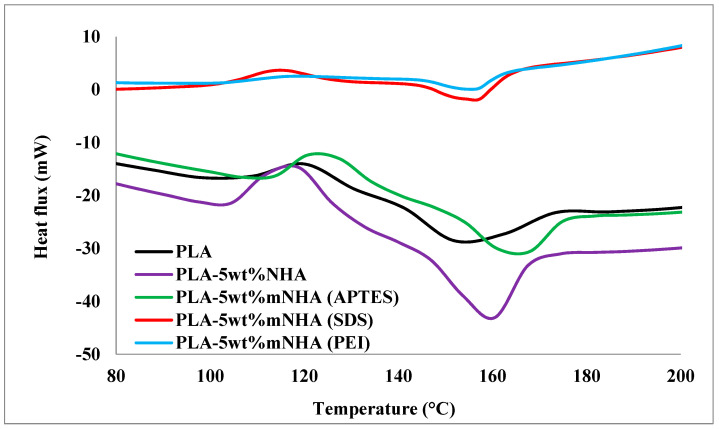
DSC curve for PLA, PLA-5wt%NHA, and PLA-5wt%mNHA treated with APTES, SDS, and PEI.

The DSC thermogram of the surface-treated and untreated nanocomposite compared to pure PLA is depicted in Figure 9, with the data tabulated in Table 4. The crystallization (T_c_) and melting (T_m_) temperatures of the nanocomposites were both shown by two peaks in the figure. Generally, the addition of NHA is expected to improve the T_c_ of the nanocomposite due to the heterogeneous nucleation of the nanofillers into the PLA matrix [14]. In contrast, the T_c_ of the PLA-5wt%NHA nanocomposite decreased by 7 °C compared to pure PLA. The T_c_ and T_m_ of the PLA-5wt%mNHA (APTES) nanocomposites were recorded as 127.4 °C and 167.9 °C, whereas T_c_ and T_m_ of PLA-5wt%mNHA (SDS) were 114.2 °C and 154.6 °C, respectively. The T_m_ of the PEI-treated nanocomposite was 154.7 °C, and the T_m_ of PLA-5wt%NHA and pure PLA was 160.5 °C and 151.8 °C, respectively. Meanwhile, adding 5wt%mNHA (APTES) into the PLA matrix caused a slight T_c_ increase compared to pure PLA, consequently delaying PLA crystallization temperature. The T_c_ of the nanocomposite was improved due to interfacial adhesion between the nanofiller and the polymer matrix. As a result, the nucleating effect of NHA was further magnified upon surface treatment with APTES. On the other hand, the addition of 5wt%mNHA (SDS) decreased T_c_ of the nanocomposite compared to both PLA-5wt%NHA and pure PLA. The enhanced brittleness of PLA-5wt%mNHA (SDS) and weak interfacial adhesion between mNHA (SDS) and PLA matrix, as well as the mechanical properties of the nanocomposite recorded in previous work, confirm this observation [1]. Furthermore, low nucleation of mNHA (PEI) due to surface treatment of NHA with PEI is linked to the disappearance of the T_c_. The nucleating effect of the nanofillers is in the order of mNHA (APTES) > NHA > mNHA (SDS) > mNHA (PEI).

Based on the data from Table 4, it is evident that the T_m_ for all nanocomposites was higher than the pure PLA, which is the consequence of nanofillers causing the hindrance in PLA’s chain mobility. Similarly, the nucleation of nanofillers and the interfacial adhesion between the nanofiller and the polymer matrix significantly impacted the T_c_ and T_m_ of nanocomposites. As a result, smoothed/improved nucleation or strong interfacial adhesion results in lower PLA chain mobility, increasing the nanocomposite’s melting point. As a result of increased interfacial adhesion, the PLA-5wt%mNHA (APTES) nanocomposite achieved the highest melting temperature led by the PLA-5wt% NHA nanocomposite due to the massive nucleating effect of NHA. In contrast, the PLA-5wt%mNHA (SDS) and PLA-5wt%mNHA (PEI) nanocomposites achieved the lowest melting temperature, similar to pure PLA. This is due to both the poor interfacial adhesion and weak nucleating effect of mNHA (SDS) and mNHA (PEI).

## 4. Conclusions

The viscoelastic characteristics and thermal stability of load-bearing bone implants is an intriguing subject to investigate. In this study, we synthesized PLA nanocomposite by blending 5 wt% NHA via melt mixing. Furthermore, in order to improve the interfacial adhesion between NHA and PLA, NHA was surface modified (mNHA) using APTES, SDS, and PEI. The FESEM analysis revealed improved interfacial adhesion between PLA matrix and mNHA (APTES), while mNHA (SDS) and mNHA (PEI) had no significant effect on interfacial adhesion. As a result, the thermal and viscoelastic properties of PLA-5wt%mNHA (APTES) nanocomposite improved while PLA-5wt%mNHA (SDS) and PLA-5wt%mNHA (PEI) deteriorated in comparison to both neat PLA and PLA-5wt%NHA. Further studies are required to assess the biocompatibility of PLA nanocomposite for load-bearing applications.

## Figures and Tables

**Table 1:** Designation of the nanocomposites.

Designation	PLA (wt%)	NHA (wt%)	mNHA (wt%)
PLA	100	-	-
PLA-5wt%NHA	95	5	-
PLA-5wt%mNHA (APTES)	95	-	5
PLA-5wt%mNHA (SDS)	95	-	5
PLA-5wt%mNHA (PEI)	95	-	5

**Table 2:** Effectiveness of surface modifiers on the dynamic mechanical properties with respect to pure PLA and PLA-5wt% NHA.

Sample	Storage Modulus (MPa)	T_g_ (°C)	Damping Parameter	C	Storage Modulus Retention (%)			
					E′_50__°C_/E′_40__°C_	E’_60__°C_/E′_40__°C_	E′_70__°C_/E′_40__°C_	E′_80__°C_/E′_40__°C_
PLA	821.58	70.24	2.04	-	99	91	1.3	0.3
PLA-5wt%NHA	1086.13	69.64	2.04	0.37	98	90	3	0.9
PLA-5wt%mNHA (APTES)	849.61	70.06	1.12	0.81	98	80	1.8	0.4
PLA-5wt%mNHA (SDS)	442.32	63.69	1.81	0.85	99	12.4	0.6	0.4
PLA-5wt%mNHA (PEI)	590.88	65.11	1.82	0.87	97	12.6	0.5	0.4

**Table 3:** Creep analysis of the nanocomposites in comparison to pure PLA.

Sample	Condition	Creep Strain	Recovery Strain	Residual Strain
PLA	T = 70 °C	0.2814	0.1940	0.0874
PLA-5wt%NHA	T = 60 °C	0.0246	0.0162	0.0084
	T = 70 °C	0.0309	0.0205	0.0104
	T = 80 °C	0.0324	0.0039	0.0285
PLA-5wt%mNHA (APTES)	T = 60 °C	0.0215	0.0144	0.0071
	T = 70 °C	0.0274	0.0157	0.0117
	T = 80 °C	0.1371	0.0290	0.1081
PLA-5wt%mNHA (SDS)	T = 70 °C	0.0275	0.0157	0.0118
PLA-5wt%mNHA (PEI)	T = 70 °C	0.0301	0.0193	0.0108

**Table 4:** Thermal properties of the surface treated nanocomposites with respect to untreated nanocomposite and pure PLA.

Samples	TGA Results					DSC Results			
	T_5%_ (°C)	T_10%_ (°C)	T_50%_ (°C)	Max T_deg_ (°C)	Remaining Weight (%)	T_c_ (°C)	Heat Flow (mW)	T_m_ (°C)	Heat Flow (mW)
PLA	332.8	339.4	360.1	367.5	2.91	125.2	−17.19	151.8	−28.53
PLA-5wt%NHA	332.8	339.4	360.1	368.9	5.29	118.8	−14.69	160.5	−43.08
PLA-5wt% mNHA (APTES)	326.9	340.5	364.9	371.5	11.09	127.4	−13.04	167.9	−30.60
PLA-5wt% mNHA (SDS)	311.5	316.4	331.7	335.4	8.68	114.2	3.66	155.6	−16.22
PLA-5wt% mNHA (PEI)	341.6	342.6	366.4	370.4	9.83	-	-	154.7	0.08

## Data Availability

Not applicable.
